# Assessing the National Cancer Institute’s SmokefreeMOM Text-Messaging Program for Pregnant Smokers: Pilot Randomized Trial

**DOI:** 10.2196/jmir.8411

**Published:** 2017-10-03

**Authors:** Lorien C Abroms, Shawn Chiang, Laura Macherelli, Leah Leavitt, Margaret Montgomery

**Affiliations:** ^1^ Milken Insitute School of Public Health George Washington University Washington, DC United States; ^2^ Department of Obstetrics and Gynecology MedStar Washington Hospital Center Washington, DC United States

**Keywords:** mHealth, text messaging, SMS, mobile phone, pregnant, smoking, quit

## Abstract

**Background:**

Automated text messages on mobile phones have been found to be effective for smoking cessation in adult smokers.

**Objective:**

This study aims to test the acceptability and feasibility of SmokefreeMOM, a national smoking cessation text-messaging program for pregnant smokers.

**Methods:**

Participants were recruited from prenatal care and randomized to receive SmokefreeMOM (n=55), an automated smoking cessation text-messaging program, or a control text message quitline referral (n=44). Participants were surveyed by phone at baseline and at 1 month and 3 months after enrollment.

**Results:**

Results indicate that the SmokefreeMOM program was highly rated overall and rated more favorably than the control condition in its helpfulness at 3-month follow-up (*P*<.01) and in its frequency of messaging at both 1-month and 3-month follow-ups (*P*<.001, *P*<.01, respectively). Despite the presence of technical problems, the vast majority of intervention participants read all program messages, and few participants unsubscribed from the program. There were no significant differences between groups on the use of extra treatment resources or on smoking-related outcomes. However, at the 3-month follow-up, some outcomes favored the intervention group.

**Conclusions:**

SmokefreeMOM is acceptable for pregnant smokers. It is recommended that SmokefreeMOM be further refined and evaluated.

**Trial Registration:**

Clinicaltrials.gov NCT02412956; https://clinicaltrials.gov/ct2/show/NCT02412956 (Archived by WebCite at http://www.webcitation.org/6tcmeRnbC)

## Introduction

Cigarette smoking in pregnancy poses serious health risks to both the pregnant woman and the fetus. It has been shown to cause adverse fetal outcomes including stillbirths, spontaneous abortions, premature births, low birthweight, and sudden infant death syndrome and has been linked to cognitive and behavioral problems in children [1,2]. It is estimated that 20% or more of low birth-weight births could be prevented by eliminating smoking during pregnancy [3].

Approximately 10% of women smoke throughout their pregnancy in the United States [4,5]. Pregnant smokers are typically younger, less educated, and more likely to be white or of Native American ancestry [6]. Barriers to quitting reported by pregnant smokers include a lack of willpower, stressful life events and relationships, and factors associated with smoking among family and friends [7]. The stigma associated with pregnancy smoking has also been reported as a barrier to treatment seeking [7].

Mobile phones and text messaging have become widespread. In the United States, 95% of all American adults own a mobile phone, and among those with a high school education or less, 92% own a mobile phone [8]. Most mobile phone owners (88%) send and receive text messages [9]. Texting is more common in younger adults than older adults, those of African American or Hispanic ethnicity compared with whites [9], and in people with Medicaid health insurance compared with other forms of private insurance [10].

Automated text messages on mobile phones have been found to be effective for smoking cessation in adult smokers [11-13]. These programs, which generally consist of interactive text messages, can mirror many of the elements of in-person counseling including goal setting and feedback, social support, and the provision of a personalized quit plan [14]. The Guide to Community Preventive Services in the United States added mobile programs for smoking cessation to its list of recommended treatments for smoking cessation [15].

Mobile phone‒based programs may be especially well suited to pregnant smokers for several reasons. Mobile phones have nearly universal penetration among women of childbearing age [9]. In addition, because of the stigma associated with smoking during pregnancy, pregnant smokers may prefer a self-help program where they seek help anonymously [16]. Furthermore, as standard in-person counseling programs fail to reach most pregnant smokers [17,18], new delivery platforms are needed.

A handful of studies have been conducted on smoking cessation in pregnancy with mobile phone‒based support [19-21]. In a smoking cessation text-messaging program with pregnant smokers enrolled in Text4baby, Abroms et al found that text messages increased self-reported quitting during pregnancy [20]. In addition, a randomized trial by Naughton et al in prenatal clinics found that those randomized to text messages reported favorable outcomes on the psychosocial mediators of quitting [21]. Finally, in a pilot, Pollak et al found support for text messages that were used to prompt a scheduled gradual reduction in smoking among pregnant smokers [19].

The current study is the first to test the acceptability and feasibility of an existing national text-messaging program, SmokefreeMOM, which is aimed at pregnant smokers. SmokefreeMOM was created by researchers at the George Washington University and has been offered as a free service by the National Cancer Institute as part of their Smokefree website since 2014. This study examines SmokefreeMOM in the context of a pilot randomized trial of patients recruited from prenatal care clinics in the greater Washington, DC, area. The results of this study are important because this study is the first formative evaluation of a program that is nationally available, and new treatments are needed aimed at pregnancy cessation.

## Methods

### Study Procedures

The study was approved by the George Washington University (GWU) Institutional Review Board in 2013. Patients were recruited from 11 obstetrics-gynecology clinics in the Washington, DC, metropolitan area between September 5, 2014, and May 25, 2016. Nine of the clinics were associated with Medstar Health, while one was part of the George Washington University Medical Faculty Associates (GWU MFA) and the other part of Capital Women’s Care. Patients were recruited in two ways. First, at Medstar Health and GWU MFA, patients were identified by searching the electronic health record (EHR) for patients who met the criteria of being pregnant and a current smoker. Once identified, these patients were sent a letter with study-related information and instructions on contacting study staff to join the study or be removed from the list. For patients who did not contact study staff, research clinical staff called patients to assess their interest in participating. In addition to this strategy, clinical providers at all study sites were made aware of the study and asked to refer their pregnant patients who smoked to the study staff. In this case, providers assessed patient interest in participating in the study, and with patient permission, provided contact information to GWU study staff. See Multimedia Appendix 1 for the CONSORT checklist for this study [22].

Research staff called patients over the phone and assessed their eligibility. Patients were eligible if they were currently pregnant, spoke and read English proficiently, had a mobile phone with unlimited text-messaging, and were currently smoking or had smoked in the past 2 weeks at the time of enrollment. If interested and eligible, participants were consented over the phone and enrolled in the study, given a baseline survey and then randomized to a study arm. Participants were followed up with a phone survey at 1 month and 3 months after enrollment. A saliva sample was also collected from participants who reported not smoking in the past 7 days at the 3-month follow-up. For saliva collection, participants were mailed a kit with instructions, a salivette, and a prepaid postage envelope for sample return. Samples that were returned were kept in a refrigerator and mailed in batches to J2 Labs (Tucson, AZ) for cotinine analysis, following methods from our earlier trials [13,20]. Participants received a US $25 gift card for completing each survey and for providing a saliva sample. All survey data were collected with the REDCap data collection tool [23].

At the start of the trial, participants were randomized to one of three groups: the control group, the SmokefreeMOM group, and the SmokefreeMOM + quitline group. Recruitment progressed slower than projected because potential participants, the majority of whom were identified through the EHR as pregnant and smoking, when screened were determined not to be pregnant or smoking. Because of these difficulties associated with recruitment and because on initial review the quitline group did not appear to be receiving quitline services at high rates, a decision was made 2 months after the start of the trial to discontinue recruitment into the SmokefreeMOM + quitline group and randomize future participants to only the SmokefreeMOM and control groups. At this time, 8 participants had been enrolled in the SmokefreeMOM + quitline group.

#### Control Group

Control group participants were texted a single text message after enrollment and were mailed self-help printed materials from the Centers for Disease Control and Prevention (CDC) on quitting smoking while pregnant [24]. The single text message provided a referral to the telephone quitline: “BeFree Study. For help quitting smoking, call the quitline and get free advice from a quit counselor. Call 1-800-784-8669 (1-800-QUIT- NOW).”

#### SmokefreeMOM

Participants randomized to the SmokefreeMOM group were enrolled in the SmokefreeMOM text messaging program by study staff and were mailed self-help materials from the CDC on quitting smoking while pregnant [24]. SmokefreeMOM is an automated, text-messaging program designed to help pregnant smokers quit smoking. It was created at GWU and incorporated into Smokefree.gov service offerings before the start of the trial. It is publicly available at the Smokefree website, but for the purposes of the trial, participants were enrolled using a trial-specific Web portal.

The text-messaging program was developed following a series of indepth interviews with pregnant smokers about their needs and preferences for smoking cessation (N=23) [25]. The program was developed based on Bandura’s social cognitive theory [26]. Messages provided advice and tips about how to quit smoking (ie, behavioral capability), social support, encouragement for quitting (ie, self-efficacy), information about the harms of smoking on a baby’s development (ie, outcome expectations of not quitting), and advice from ex-smokers (ie, self-efficacy). While most messages were one-way messages, some provided opportunities for two-way interaction. These included interactive surveys that assessed readiness to quit and progress in quitting. Interaction also occurred through keywords. Participants were told in messages that they could text keywords to receive additional messages or unsubscribe from the service. Participants could text at any time the keyword SMOKED if they had experienced a lapse, CRAVE if they were craving a cigarette, DATE to reset their quit date, and FACT to get a fact and learn about the harms of smoking. See Table 1 for sample messages.

Following a series of messages timed to enrollment, messages were scheduled around a participant’s quit date and baby’s due date, which were entered in as part of the enrollment process. Depending on dates entered, users received approximately 3-6 messages/day with a higher volume of messages around the quit date and around the baby’s due date. While the study ended 3 months after enrollment, program messages were designed to last 6 months after the quit date and 3 months after the baby’s due date. As the program was publicly available, those randomized to receive SmokefreeMOM could continue to receive program messages after the study’s completion.

#### SmokefreeMOM + Quitline

Participants randomized to the SmokefreeMOM + Quitline group received the same intervention as the SmokefreeMOM group with the addition of the opportunity to be enrolled in state quitline services at the time of enrollment. With permission from the participant, research staff fax enrolled participants in the SmokefreeMOM + Quitline group in quitline services. Staff faxed the name and phone number of participants to the quitline from their corresponding state (ie, District of Columbia [DC], Maryland [MD], and Virginia [VA]). Once the fax referral was received, quitline staff operated under their usual service protocol and made multiple attempts to reach participants and enroll them in quitline counseling and other services. In MD, during the study period, participants were offered an additional financial incentive for engaging in phone counseling by quitline staff. Besides this, quitline services were comparable across MD, DC, and VA, with 10 proactive counseling calls provided for pregnant smokers.

### Measures and Analysis

Measures for this study were collected on the baseline, 1-month, and 3-month follow-up surveys.

The baseline survey captured information on participant demographics, mobile phone and social media use, and smoking behavior. Nicotine dependence was measured on the baseline survey with the Fagerstrom Test for Cigarette Dependence (FTCD). Scores range from 0-10, with a score of 6 or more indicating the highest level of dependence [27,28].

#### Program Acceptability and Feasibility

Acceptability was measured at 1-month and 3-month follow-up by questions that asked participants to rate their agreement with statements about the text programs (eg, “The text(s) was/were helpful in getting me to try to quit,” “I would recommend the text(s) to a friend who was pregnant and smoking,” and “The texts were a trigger and made me want to smoke”). These statements were rated on a 5-point Likert scale from completely disagree (1) to completely agree (5). Acceptability was also measured by having participants rate their satisfaction with the number of texts received (too many, just the right number, or too few). For the intervention group, participants reported on the proportion of text messages read (100%, 75%, or 50% or less). Intervention group participants were also asked in an open-ended format what they liked and did not like about the program. Likes were grouped into the following categories: the content of texts, social support provided by texts, reminders about quitting, encouragement about quitting, interactive tools, general liking, and other. Dislikes were coded into the following categories: nothing, technical problems experienced, message frequency, the content of texts, texts were a trigger, and other. Participants could indicate more than one like or dislike.

**Table 1:** Examples of text messages from SmokefreeMOM.

	Message	Sending algorithm
Welcome message	Welcome to Smokefree Moms! Quitting smoking is the best thing for you and your baby! Up to 6 msgs/day. Msg&data rates may apply. Reply STOP to opt-out, HELP for info.	Triggered by enrollment
Pre-quit advice	SFM: Almost the big day! Throw any remaining cigs in the trash before you go to bed tonight. Get plenty of sleep. Wake up feeling fresh and ready!	Quit date -1, 10 a.m.
Message from peer ex-smoker	SFM/Lea: It was really hard for me to give up my morning cig. When is going to be hardest for you? Text 1 for when you wake in the morning, 2 for after you eat, and 3 after the kids go to bed.	Quit date -2, 12 p.m.
Quit day	SFM: Just 20 minutes after you stop smoking your blood circulation beings to improve—quitting will improve blood flow to your developing baby.	Quit date, 5 p.m.
Postquit advice	SFM: Feeling cranky? It will pass. Your body is in nicotine withdrawal. Text CRAVE at any time to help with a craving.	Quit date +2, 12 p.m.
Quit status check-in	How is it going? Have you smoked a cig, even a drag, in the past week? Text YES or NO	Quit date + 7, 2 p.m.
Baby tips	BabyTip: At 26 weeks, your baby has fingernails and may be 14 inches long from head to feet.	Due date - 94
DATE	SFM: Let’s set your new quit date for mm/dd/yy. Reply 1 to accept this date or send us another date in the next 2 weeks in MMDDYY format (051215 for May 12th).	User texts in DATE
GAME	SFM: Adults have 206 bones. When babies are born, how many bones do they have? A) 150 B) 200 C) 300. Reply with letter of your response.	User texts in GAME
FACT	Smoking speeds up heart rate and increases blood pressure. Every puff increases the carbon monoxide in your blood making less oxygen available to baby.	User texts in FACT
CRAVE	To calm self, breathe in through nose and stretch arms up to the sky. Breathe out through mouth and bring arms back down. For more, reply TIP or GAME.	User texts in CRAVE

In addition, for the intervention group, acceptability was measured by a retrospective review of computer records of their engagement with the program. Engagement was assessed by measuring participant responses to a series of quit status check-ins (eg, “SFM: How is it going? Have you smoked a cig, even a drag, in the past week? Text YES or NO”). Over 3 months, there were potentially 20 check-ins, depending on the scheduled quit date. The number of total replies to the check-ins was tabulated and averaged across participants. While other types of engagement would also be of interest (eg, keyword use), the research team did not have access to this data. In addition, dissatisfaction with the program was measured by examining whether participants unsubscribed from the program. The proportion of SmokefreeMOM participants who texted STOP, a keyword for unsubscribing, was calculated at 1 month and 3 months.

Feasibility was assessed by asking all participants about the presence of technical problems related to the text messages. Technical problems were assessed using a combination of two survey items on the 1-month and 3-month surveys to capture the full extent of problems. Participants were asked whether they experienced any technical problems since enrolling in the study. If they answered “yes,” they were coded as having had a technical problem. In addition, participants were coded as having had a technical problem if they answered “no” on this survey item but reported technical problems in a separate open-ended question about what they did not like about the program.

#### Use of Treatments and Resources for Quitting

Use of treatments and resources for quitting was assessed with a question at 1-month follow-up that asked, “Since enrolling in this study, did you use any of the following to help you quit?” Participants were read the following options: Telephone help/quitline, one-on-one counseling, study-provided self-help materials, other self-help materials, quit smoking website, e-cigarettes, text messages from this study, text from another program, medication, other, and none of the above. Participants could indicate use of more than one treatment or resource.

#### Smoking-Related Outcomes

Smoking was measured by assessing 7-day biochemically confirmed point prevalence abstinence (PPA) at the 3-month follow-up, defined as a self-report of no smoking in the past 7 days on the 3-month survey and a cotinine level ≤13 ng/mL from the saliva sample [29]. Other outcomes assessed at 1-month and 3-month follow-up consisted of self-report of abstinence (7 days and 30 days), consecutive days quit, and 24-hour quit attempts. In addition, cigarettes smoked per day were measured, and number of cigarettes smoked was compared to baseline numbers by calculating a change score for each participant. Finally, self-efficacy was measured using the item, “How confident are you that you can quit smoking during this pregnancy?” Self-efficacy was measured on a 7-point scale ranging from “not at all” to “extremely.” Self-efficacy levels at 1 month and 3 months were compared to baseline levels by calculating a change score for each participant.

### Analysis

Because very few participants in the SmokefreeMOM + Quitline group reported receiving quitline services (n=2) and because the group was small, a decision was made to combine the SmokefreeMOM + Quitline group (n=8) with the SmokefreeMOM group (n=47) and to compare these combined groups (referred to as “the intervention group”) to the control group (n=44). Before combining groups, baseline demographic characteristics between the SmokefreeMOM + Quitline group and the SmokefreeMOM group were compared with no major differences observed.

Next, baseline demographic differences between the intervention and control groups were tested with independent *t* tests or chi-square tests. At 1-month and 3-month follow-up, differences between outcomes in the intervention and control groups were tested with independent *t* tests or chi-square tests. For dichotomous smoking outcomes (eg, 7-day and 30-day PPA), missing data were imputed as smoking. Where baseline differences were observed between groups, unadjusted and adjusted regression models were run to control for differences. Results were found to be similar for the unadjusted and adjusted models; therefore, unadjusted models are presented. Analyses were conducted in SPSS v. 22.0.

**Figure 1:**
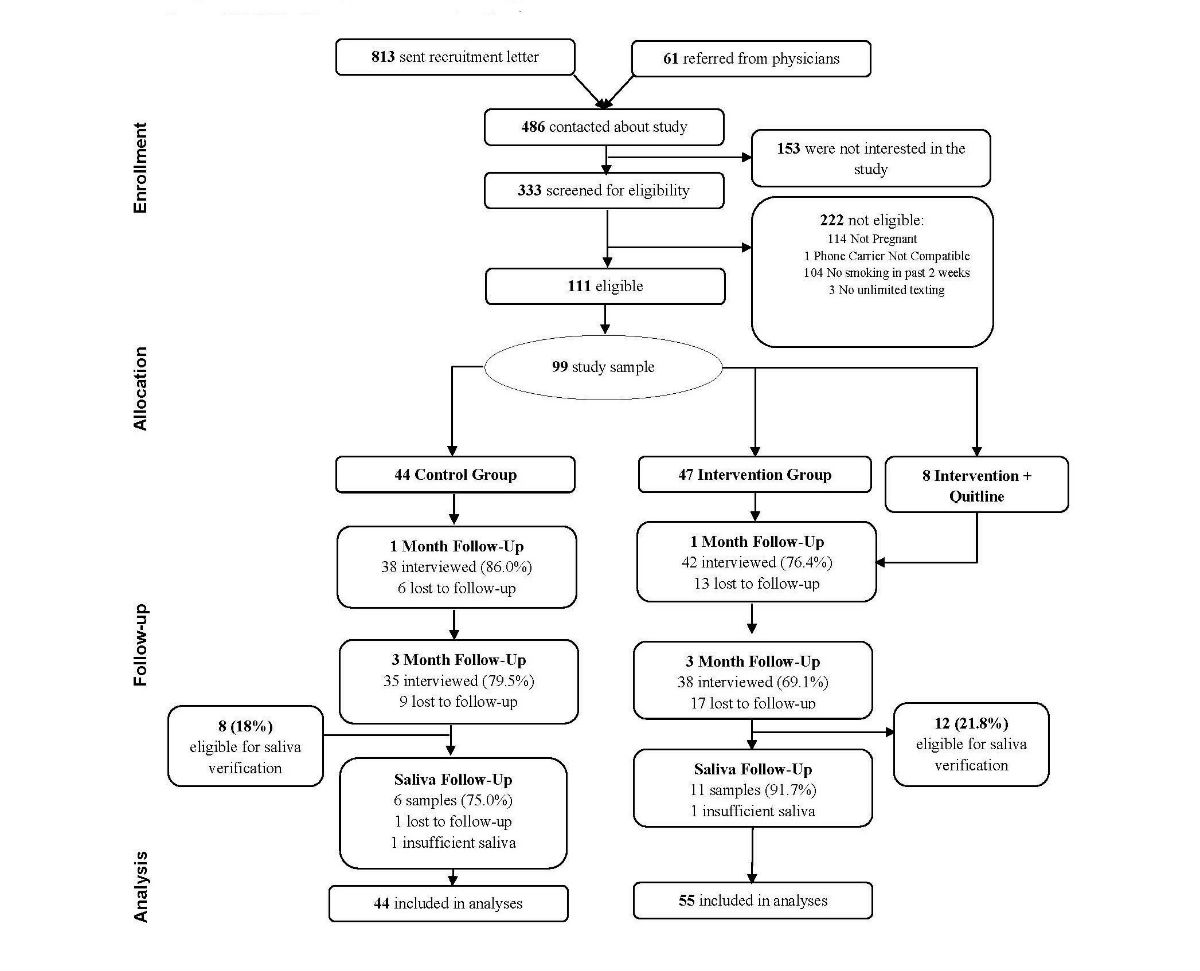
Participant enrollment and follow-up.

**Table 2:** Demographic characteristics of participants.

Baseline characteristics		Intervention, n=55	Control, n=44	Total, N=99
Age, mean (SD)		27.18 (4.98)	28.25 (4.78)	27.66 (4.90)
**Race/ethnicity, n (%)**				
	White	32 (58.18)	23 (52.27)	55 (55.56)
	African American	22 (40.00)	18 (40.91)	40 (40.40)
	Other	1 (1.82)	3 (6.82)	4 (4.04)
**Education^a^, n (%)**				
	12th grade or less with no high school diploma	17 (30.91)	9 (20.45)	26 (26.26)
	High school graduate or equivalent	15 (27.27)	11 (25.00)	26 (26.26)
	Some college	20 (36.36)	10 (22.73)	30 (30.30)
	Associates or higher	3 (5.45)	14 (31.82)	17 (17.17)
**Employment status, n (%)**				
	Part time	6 (10.91)	7 (15.91)	13 (13.13)
	Full time	19 (34.55)	15 (34.09)	34 (34.34)
	Not at all	30 (54.55)	22 (50.00)	52 (52.52)
**Household income in US $, n (%)**				
	˂$15,000	22 (40.00)	18 (40.91)	40 (40.40)
	$15,001-$30,000	14 (25.45)	13 (29.55)	27 (27.27)
	$30,001-$47,099	8 (14.55)	5 (11.36)	13 (13.13)
	≥$47,100	10 (18.18)	7 (15.91)	17 (17.17)
**Marital status, n (%)**				
	Single, never married	26 (47.27)	20 (45.45)	46 (46.46)
	Living with significant other	16 (29.09)	12 (27.27)	28 (28.28)
	Married	11 (20.00)	9 (20.45)	20 (20.20)
	Divorced/separated, widowed	2 (3.64)	3 (6.82)	5 (5.05)
Gestational age (in weeks), mean (SD)		22.15 (9.54)	20.51 (10.49)	21.42 (10.17)
Mobile phone ownership, n (%)		49 (89.09)	42 (95.45)	91 (91.92)
Social media: Facebook user, n (%)		49 (89.09)	36 (81.82)	85 (85.86)
Texts per day, n (%)		64.36 (113.61)	115.73 (334.24)	87.19 (238.31)
Cigarettes per day at baseline, mean (SD)		6.89 (4.86)	6.69 (5.38)	6.80 (5.07)
FTCD (0-10), mean (SD)		2.68 (2.15)	2.68 (2.24)	2.68 (2.17)
Baseline self-efficacy (1-7), mean (SD)		4.95 (1.74)	4.59 (1.86)	4.79 (1.79)
Smoked an e-cig in the past 30 days, n (%)		12 (21.82)	6 (13.64)	18 (18.18)
Alcohol consumption in past 30 days (≥1 drink), n (%)		5 (9.09)	3 (6.82)	8 (8.08)

^a^*P*<.01.

## Results

As shown in Figure 1, 333 participants were screened for eligibility and 111 were found to be eligible. Reasons for ineligibility were not being pregnant (n=114), not smoking in the past 2 weeks (n=104), not having unlimited texting (n=3), and not having a phone carrier compatible with SmokefreeMOM (n=1). Of those eligible, 99 participants enrolled in the study (89.2%, 99/111). We randomized 44 participants to the control group and 55 participants to the combined intervention group; initially 47 were in the SmokefreeMOM group and 8 in the SmokefreeMOM + Quitline group. Follow-up rates were 80.8% for 1-month follow-up and 73.7% for 3-month follow-up. The majority (85%, 17/20) of eligible participants returned a valid saliva sample to biochemically verify smoking status. Differences in follow-up rates were not statistically different between groups.

### Participant Characteristics

Participant characteristics are presented in Table 2. Participants were on average 27.66 (SD 4.90) years old, predominantly white non-Hispanic (56%, 55/99) and African American (40%, 40/99), and on average, 21.42 weeks pregnant (SD 10.17) at the time of the enrollment. Over half the sample (53%, 52/99) had a high school diploma or less, and over half were not employed (53%, 52/99). The majority of the sample had a household income of US $30,000 a year or less (68%, 67/99). At the time of enrollment, participants smoked an average of 6.80 (SD 5.07) cigarettes per day and had an FTCD score of 2.68 (SD 2.17). About 18% (18/99) of the sample reported smoking an e-cigarette in the past 30 days at baseline. On average, participants sent or received 87.19 (SD 238.31) text messages per day prior to enrolling in the study. Intervention and control group participants were similar across all variables except for education, where more participants in the control group had an associate’s degree or higher and fewer with a high school diploma or less (*P*<.01).

### Program Acceptability and Feasibility

As shown in Table 3, while participants in both groups rated the program favorably on a 5-point scale, there was a trend for the intervention group to provide higher ratings. The intervention group gave higher overall ratings to the program for the degree to which they would recommend it to a friend and for its helpfulness compared with the control group. Differences between groups were significantly different at 3-month follow-up for helpfulness (*P*=.003), with intervention group participants agreeing at a higher level (4.00) on average that the program was helpful compared with the control group (3.12). While the question was not asked of the control group, participants in the intervention group agreed at low levels that the program texts were a trigger for smoking: 1.76 (SD 1.22) for 1 month and 1.56 (SD 0.97) for 3 months. The acceptability of message frequency was found to be significantly different between intervention and control at both 1 month (*P*<.001) and 3 months (*P*=.002) with most intervention participants indicating that the number of texts was just the right number. At 1-month follow-up, 78% (31/40) of intervention participants reported that the number of text messages was just right compared with 52% (15/29) of the control participants. Control participants were more likely to say that texts were too few. At both time points, a high proportion of intervention participants reported reading all (100%) of the text messages: at 1-month follow-up, 78% (31/40) of participants reported reading all of the text messages and at 3 months, 82% (22/27) of participants reported reading all of the text messages.

Participants in the intervention group provided a variety of reasons for liking the program. The most common reason volunteered was that they liked the program for the content or information provided in the program (eg, information on the harms of smoking to the baby). This reason was followed by the social support provided (eg, support from the stories about other people’s quitting), the messages served as reminders, the messages provided encouragement, the help provided by the interactive tools such as the keywords GAME and FACT, general liking and finally, for other reasons (eg, the timing of the messages, the confidence for quitting from the messages). Participants also provided a variety of reasons for not liking the program including, in order of prevalence, nothing, the technical problems associated with receiving the program, the message frequency (eg, too many messages), the content of the messages (eg, messages were too congratulatory, information was repetitive), the texts were a trigger for smoking, and other reasons (eg, needed a human element, the timing was not good).

Based on computer records of intervention program use, few participants in the intervention group unsubscribed from the program with 2 participants unsubscribing by the 1-month follow-up and 1 additional participant unsubscribing by the 3-month follow-up. For engagement, intervention participants responded 3.49 times (SD 4.02) on average to the quit-status check-ins over the 3-month period, and 69% (38/55) of participants replied to the status check-in at least once. On average, participant replies lasted 28.96 days (SD 35.09) after enrollment.

Feasibility was measured by the presence of technical programs: 29% (12/42) of participants reported technical problems at 1-month follow-up and 13% (5/38) of participants reported technical problems at 3-month follow-up. Most of these problems involved not receiving the messages, not being able to get responses when they replied back to the program or used a keyword.

### Use of Treatment and Resources

The use of treatments and resources for quitting at 1-month follow-up did not vary significantly across groups, with the exception of text messages from the study (*P*<.01), which was by design (see Table 4). Aside from the study-related text messages, over 20% of both groups reported using the study provided self-help materials. The control group did report using at higher rates other self-help materials (18% compared with 7%) (not significant) and “other” treatments and resources (21% compared with 2%) (*P*<.01). Other resources used by the control group included counseling from family members and friends, willpower, and eating sunflower seeds and candy. Both groups used one-on-one counseling (21%, 17/80), called the quitline (9%, 7/80), and used websites at similar rates (10%, 8/80).

**Table 3:** Program acceptability and feasibility.

Acceptability and feasibility			1-month follow-up			3-month follow-up		
			Intervention, n=42	Control, n=38	*P*	Intervention, n=38	Control, n=35	*P*
I would recommend the text(s) to a friend who was pregnant and smoking, mean (SD)			4.39 (1.09)	3.84 (1.42)	.07	4.32 (1.09)	3.74 (1.56)	.08
The text(s) was/were helpful in getting me to try to quit smoking, mean (SD)			3.70 (1.29)	3.07 (1.51)	.06	4.00 (1.09)	3.12 (1.65)	.003^a^
The texts were a trigger and made me want to smoke, mean (SD)			1.76 (1.22)	‒	‒	1.56 (0.97)	‒	‒
**Number of text messages, n (%)**					<.001^a^			.002^a^
	Too many		8 (20.00)	1 (3.45)		5 (17.24)	2 (6.06)	
	Just the right number		31 (77.50)	15 (51.72)		20 (68.97)	12 (36.36)	
	Too few		1 (2.50)	13 (44.83)		4 (13.79)	19 (57.58)	
**Proportion of text messages read, n (%)**								
	100%		31 (77.50)	–	–	22 (81.48)	–	–
	75%		9 (22.50)	–	–	3 (11.11)	–	–
	≤50%		0 (0.00)	–	–	2 (7.41)	–	–
**Liked about the program^b^, n (%)**								
	Content/information (eg, on harms of smoking)		18 (42.86)			13 (34.21)		
	Social support/other’s people’s quitting stories		6 (14.29)			3 (7.89)		
	Reminders		6 (14.29)			2 (5.26)		
	Encouragement		4 (9.52)			2 (5.26)		
	Interactive tools (eg, GAME, FACT)		4 (9.52)	–	–	5 (13.16)	–	–
	General liking		4 (9.52)			5 (13.16)		
	Other (eg, timing, confidence, made accountable)		4 (9.52)			5 (13.16)		
**Disliked about the program^b^, n (%)**								
	Nothing		23 (54.76)			20 (52.63)		
	Technical problems		6 (14.29)			2 (5.26)		
		Message frequency (eg, too frequent)	3 (7.14)			2 (5.26)		
		Content (eg, too congratulatory, repetitive)	2 (4.76)			1 (2.63)		
		Text as trigger	2 (4.76)	–	–	0 (0.00)	–	–
		Other (needed human element, timing)	4 (9.52)			0 (0.00)		
	Total replies to quit day check-ins, mean (SD)		2.74 (3.29)	–	–	3.49 (4.25)	–	–
	Unsubscribed from the program		2 (4.76)	–	–	1 (2.63)	–	–
	Experienced any technical problems		12 (28.57)	2 (5.26)	.006^a^	5 (13.15)	2 (5.71)	.28

^a^*P* values are statistically significant.

^b^Participants could select multiple reasons.

**Table 4:** Use of treatments and resources for quitting at 1 month.

Quit treatment and resources^a^	1 month, n (%)		
	Intervention, n=42	Control, n=38	Total
Telephone help/quitline	4 (9.52)	3 (7.89)	7 (8.75)
One-on-one counseling	9 (21.43)	8 (21.05)	17 (21.25)
Study-provided self-help materials	10 (23.81)	9 (23.68)	19 (23.75)
Other self-help materials	3 (7.14)	7 (18.42)	10 (12.50)
Quit smoking website	4 (9.52)	4 (10.53)	8 (10.00)
E-cigarettes	6 (14.29)	3 (7.89)	9 (11.25)
Text messages from this study^b^	31 (73.81)	4 (10.53)	35 (43.75)^c^
Text from another program	1 (2.38)	1 (2.63)	2 (2.50)
Use of medication	1 (2.38)	0 (0.00)	1 (1.25)
Others^b^	1 (2.38)	8 (21.05)	9 (11.25)^c^
None of the above	5 (11.90)	9 (23.68)	14 (17.50)

^a^Responses are not mutually exclusive.

^b^Statistical significance.

^c^*P*<.01.

**Table 5:** Smoking-related outcomes by time period.

	1-month follow-up		3-month follow-up	
	Intervention, n=55	Control, n=44	Intervention, n=55	Control, n=44
Biochemically confirmed 7-day PPA^a^, n (%)	‒	‒	8 (14.55)	4 (9.09)
Not smoked in past 7 days^a^, n (%)	11 (20.00)	7 (15.90)	14 (25.45)	8 (18.18)
Not smoked in past 30 days^a^, n (%)	6 (10.91)	1 (2.27)	9 (16.36)	7 (15.91)
Consecutive days quit, mean (SD)	9.58 (11.17)	5.95 (8.21)	27.24 (32.19)	17.85 (23.24)
Quit attempt (≥24 hours), n (%)	32 (58.18)	22 (50.00)	26 (47.3)	30 (68.2)
Cigarettes smoked/day, mean (SD)	3.95 (4.85)	3.46 (3.34)	2.82 (3.83)	4.26 (4.80)
Change in cigarette per day from baseline, mean (SD)	-2.74 (3.70)	-2.86 (4.79)	-3.15 (3.68)	-2.61 (4.96)
Self-efficacy (1-7), mean (SD)	5.44 (1.83)	5.28 (1.71)	5.41 (1.76)	5.03 (1.70)
Change in self-efficacy, mean (SD)	0.39 (1.63)	0.34 (1.80)	0.34 (1.99)	0.39 (2.00)

^a^Missing data were imputed to indicate smoking.

### Smoking Outcomes

Table 5 summarizes the unadjusted smoking-related outcomes. Adjusting the dichotomous smoking outcomes for baseline differences in education did not significantly change the results. At both 1-month and 3-month follow-up, there were no significant differences in any of the smoking-related outcomes between groups, including biochemically confirmed 7-day PPA, self-reported 7-day and 30-day abstinence, consecutive days quit, quit attempts, and changes in cigarettes smoked/day. Though not significantly different, results were favorable to the intervention group at 3 months on biochemically confirmed 7-day PPA with 15% (8/55) of the intervention group reporting abstinence compared with 9% (4/44) of the control group and on consecutive days quit with the intervention group reporting 27.24 (SD 32.19) days quit compared with 17.85 (23.24) days quit in the control group. Change in self-efficacy was not significantly different between groups.

## Discussion

### Principal Findings

This study aimed to assess the acceptability and feasibility of an existing national text-messaging program, SmokefreeMOM, with pregnant smokers recruited from prenatal clinics. Results indicate that SmokefreeMOM was rated highly and more favorably than a control condition that consisted of a single text message in its helpfulness at 3-month follow-up and in its frequency of messaging at both time points. Among the intervention group participants, messages were read at high rates and participants unsubscribed from the program at low rates. Nonetheless, almost 30% of participants experienced some technical problems with the program during the study period, largely related to not being able to get responses from the automated system after replying to queries or sending in keywords. There were no significant differences between groups on use of extra treatment resources or on smoking-related outcomes, though some outcomes favored the intervention group at 3-month follow-up.

Consistent with findings of other studies of text messaging for smoking cessation in pregnant women [19-21], it was encouraging to find that overall the program was rated favorably. SmokefreeMOM participants stated that the number of messages sent was appropriate and messages were read at high rates. They noted that they liked the program for several reasons including the information provided by the messages such as the harms of smoking to the baby, the social support from the program, and the constant reminders about quitting. Participants also noted some negative aspects of the program. A couple of participants noted that program messages were a trigger for smoking. While this had been reported in other text-messaging programs for smoking cessation [16,30], a public health intervention should not have deleterious effects on any of its participants, and the possible triggering caused by messages is worthy of future investigation. In addition, participants noted that they experienced technical problems. Still, despite these technical problems, the majority of participants replied to the status check-in at least once during the study period, and on average, participants replied 3 times. Health promotion programs that stimulate interaction and engagement have generally been found to be more likely to result in behavior change [31]. Given the difficulty associated with engaging pregnant smokers [7,18], the findings for interaction are encouraging for the SmokefreeMOM program.

It should also be noted that while the SmokefreeMOM program was rated favorably on most measures, there were no significant differences between SmokefreeMOM and the control condition on likelihood of recommending the program to a friend. This may imply that one text message with a quitline referral may be helpful compared with currently available services in prenatal care and that a fully developed program like SmokefreeMOM may be unnecessary. As prior studies have not used a lower intensity text-messaging program as their control [19-21], the utility of such an intervention based on a single text remains an open question.

The presence of technical problems in interacting with the system is also a lesson. Almost a third of participants reported technical problems, primarily related to replying to program messages. Though the program was tested prior to the launch of the study, it was not tested continuously during the study, and study staff was not aware of these problems until after study completion. To avoid technical problems, future programs should check the proper functioning of the system not only initially but repeatedly throughout the study period. It remains an open question whether the same program without technical problems would have resulted in higher levels of engagement and more favorable smoking-related outcomes.

The study was not powered to detect differences in smoking-related outcomes, and unlike prior studies [19-20], none were detected. As most indicators of acceptability are promising and the technical problems encountered have been resolved, future studies may wish to investigate the efficacy of SmokefreeMOM with larger samples of pregnant smokers. It remains unclear whether such programs are helpful in the context of prenatal care where other types of assistance may be readily available, though a prior study indicates that it may be promising [21].

One final finding of note was that few participants—9% (7/80) of all participants—reported receiving extra treatment help in the form of help from the quitline. This was in spite of a clear effort to get all participants to call the quitline. For the control group, the only text message they received was a referral to the quitline with the number provided on their phone. For the intervention group, text messages repeatedly referred participants to the quitline, including every time they indicated that they were having difficulty quitting. Additionally, 8 participants who were later included as part of the intervention group were connected (n=8) via fax enrollment to the quitline. Of these, only 2 reported receiving quitline services. This low level of use of quitline services use may indicate that this service is not appealing or not congruent with the lives of pregnant smokers. This may be because for low-income smokers, quitline services may consume almost a third of mobile phone talk minutes [32]. As quitlines remain a dominant public health strategy for pregnant smokers, this finding warrants further exploration and may point to the need to develop novel services that better fit with communication preferences of pregnant smokers [9].

### Strengths and Limitations

A main strength of this study is that this study is the first evaluation of a program that is nationally available for pregnant smokers—a group that is high-risk, underserved, and in need of new treatments. Other strengths include the use of a control group, biochemical verification of self-reported smoking status, and overall good follow-up rates.

Limitations include that recruitment was a challenge for this study. The primary mechanism for identifying potential participants was by generating a list of potential participants using the pregnant and smoking fields in the EHR. The majority of potential participants identified and later screened were found to not be pregnant or smokers. This points to the limitations of relying on EHR records for recruitment. In addition, because of difficulties with recruitment, we discontinued enrollment in one of the planned groups of the trial (SmokefreeMOM + Quitline group) 2 months after the start of the trial. Participants from this group were fax enrolled in quitline services (n=8) and may have received additional services that shaped their rating of the program and smoking-related outcomes. However, as only 2 participants from this group received counseling services from the quitline, the effect of the additional quitline service is likely limited. Another limitation is that while the intervention was aimed at pregnancy cessation, by 3-month follow-up some women gave birth during the study period (n=20: 7 in intervention and 13 control). As birth of a baby is a significant risk factor for smoking relapse, it remains unclear what the impact of the birth was on study outcomes. Additionally, as noted earlier, the SmokefreeMOM program experienced technical problems during the study period, which may have minimized the effect of the intervention. Furthermore, the study results may not be generalizable to all pregnant smokers as participants had the following characteristics: they had disclosed their smoking status to their medical provider, were from a mid-Atlantic metropolitan area, and on average were 21.42 weeks pregnant.

### Conclusions

The findings of this study show that a text-messaging program that makes use of interactive text messages timed around the quit date and a baby’s due date is acceptable to pregnant smokers. Given the evidence for the efficacy of text messaging for smoking cessation in adult smokers [11-13] and emerging evidence in pregnant smokers [19-21], it is recommended that SmokefreeMOM be further refined and a future study be designed to evaluate whether this free and readily available resource can promote cessation in pregnant smokers.

## Appendix

Multimedia Appendix 1 CONSORT-EHEALTH checklist V1.6.1 [32].

## Abbreviations

CDC: Centers for Disease Control and Prevention
EHR: electronic health record
FTCD: Fagerstrom Test for Cigarette Dependence
GWU MFA: George Washington University Medical Faculty Associates
GWU: George Washington University
NCI: National Cancer Institute
PPA: point prevalence abstinence

## References

[1] Clinical Practice Guideline Treating Tobacco Use and Dependence 2008 Update Panel‚ Liaisons‚Staff (2008). A clinical practice guideline for treating tobacco use and dependence: 2008 update. A U.S. Public Health Service report. Am J Prev Med.

[2] Pineles BL, Hsu S, Park E, Samet JM (2016). Systematic Review and Meta-Analyses of Perinatal Death and Maternal Exposure to Tobacco Smoke During Pregnancy. Am J Epidemiol.

[3] Office of the Surgeon General (1990). Health benefits of smoking cessation: a report of the Surgeon General.

[4] Centers for Disease Control and Prevention (2011). Indicator for whether mother smoked during the last three months of pregnancy.

[5] Curtin SC, Matthews TJ (2016). Smoking Prevalence and Cessation Before and During Pregnancy: Data From the Birth Certificate, 2014. Natl Vital Stat Rep.

[6] Centers for Disease Control and Prevention (2011). Vital signs: current cigarette smoking among adults aged ≥18 years--United States, 2005-2010. MMWR Morb Mortal Wkly Rep.

[7] Ingall G, Cropley M (2010). Exploring the barriers of quitting smoking during pregnancy: a systematic review of qualitative studies. Women Birth.

[8] Pew Research Center (2017). Mobile fact sheet.

[9] Smith A How Americans use text messaging.

[10] PricewaterhouseCoopers (2010). Healthcare Unwired: New business models delivering care anywhere.

[11] Free C, Knight R, Robertson S, Whittaker R, Edwards P, Zhou W, Rodgers A, Cairns J, Kenward MG, Roberts I (2011). Smoking cessation support delivered via mobile phone text messaging (txt2stop): a single-blind, randomised trial. Lancet.

[12] Whittaker R, McRobbie H, Bullen C, Rodgers A, Gu Y (2016). Mobile phone-based interventions for smoking cessation. Cochrane Database Syst Rev.

[13] Abroms L, Boal A, Simmens S, Mendel J, Windsor R (2014). A randomized trial of Text2Quit: a text messaging program for smoking cessation. Am J Prev Med.

[14] Abroms L, Padmanabhan N, Evans W (2012). Using Mobile Phones for Health Promotion. eHealth Applications: Promising Strategies for Behavior Change.

[15] Guide to Community Preventive Services (2011). Increasing tobacco use cessation: mobile phone-based interventions.

[16] Naughton F, Jamison J, Sutton S (2013). Attitudes towards SMS text message smoking cessation support: a qualitative study of pregnant smokers. Health Educ Res.

[17] McGowan A, Hamilton S, Barnett D, Nsofor M, Proudfoot J, Tappin DM (2010). 'Breathe': the stop smoking service for pregnant women in Glasgow. Midwifery.

[18] Windsor R, Woodby L, Miller T, Hardin M (2011). Effectiveness of Smoking Cessation and Reduction in Pregnancy Treatment (SCRIPT) methods in Medicaid-supported prenatal care: Trial III. Health Educ Behav.

[19] Pollak KI, Lyna P, Bilheimer A, Farrell D, Gao X, Swamy GK, Fish LJ (2013). A pilot study testing SMS text delivered scheduled gradual reduction to pregnant smokers. Nicotine Tob Res.

[20] Abroms L, Johnson P, Leavitt L (2017). Am J Prev Med.

[21] Naughton F, Cooper S, Foster K, Emery J, Leonardi-Bee J, Sutton S, Jones M, Ussher M, Whitemore R, Leighton M, Montgomery A, Parrott S, Coleman T (2017). Large multi-centre pilot randomized controlled trial testing a low-cost, tailored, self-help smoking cessation text message intervention for pregnant smokers (MiQuit). Addiction.

[22] Eysenbach G, CONSORT-EHEALTH Group (2011). CONSORT-EHEALTH: improving and standardizing evaluation reports of Web-based and mobile health interventions. J Med Internet Res.

[23] Harris PA, Taylor R, Thielke R, Payne J, Gonzalez N, Conde JG (2009). Research electronic data capture (REDCap)--a metadata-driven methodology and workflow process for providing translational research informatics support. J Biomed Inform.

[24] Centers for Disease Control and Prevention (2016). Smoking During Pregnancy.

[25] Lin M, Abroms L (2015). Unpublished report.

[26] Bandura A (1986). Social foundations of thought and action: a social cognitive theory.

[27] Fagerström K (2012). Determinants of tobacco use and renaming the FTND to the Fagerstrom Test for Cigarette Dependence. Nicotine Tob Res.

[28] Heatherton T, Kozlowski L, Frecker R, Fagerström KO (1991). The Fagerström Test for Nicotine Dependence: a revision of the Fagerström Tolerance Questionnaire. Br J Addict.

[29] Hegaard HK, Kjaergaard H, Møller LF, Wachmann H, Ottesen B (2007). Determination of a saliva cotinine cut-off to distinguish pregnant smokers from pregnant non-smokers. Acta Obstet Gynecol Scand.

[30] Douglas N, Free C (2013). 'Someone batting in my corner': experiences of smoking-cessation support via text message. Br J Gen Pract.

[31] Yardley L, Spring BJ, Riper H, Morrison LG, Crane DH, Curtis K, Merchant GC, Naughton F, Blandford A (2016). Understanding and Promoting Effective Engagement With Digital Behavior Change Interventions. Am J Prev Med.

[32] Bernstein SL, Rosner J, Toll B (2016). Cell Phone Ownership and Service Plans Among Low-Income Smokers: The Hidden Cost of Quitlines. Nicotine Tob Res.

